# Osteoblast CFTR Inactivation Reduces Differentiation and Osteoprotegerin Expression in a Mouse Model of Cystic Fibrosis-Related Bone Disease

**DOI:** 10.1371/journal.pone.0080098

**Published:** 2013-11-13

**Authors:** Michael S. Stalvey, Katrina L. Clines, Viktoria Havasi, Christopher R. McKibbin, Lauren K. Dunn, W. Joon Chung, Gregory A. Clines

**Affiliations:** 1 Department of Pediatrics, University of Alabama at Birmingham, Birmingham, Alabama, United States of America; 2 Department of Medicine, University of Alabama at Birmingham, Birmingham, Alabama, United States of America; 3 Department of Medicine, University of Virginia, Charlottesville, Virginia, United States of America; 4 Department of Neurobiology, University of Alabama at Birmingham, Birmingham, Alabama, United States of America; 5 Veterans Administration Medical Center, Birmingham, Alabama, United States of America; University of Duisburg-Essen, Germany

## Abstract

Low bone mass and increased fracture risk are recognized complications of cystic fibrosis (CF). CF-related bone disease (CFBD) is characterized by uncoupled bone turnover—impaired osteoblastic bone formation and enhanced osteoclastic bone resorption. Intestinal malabsorption, vitamin D deficiency and inflammatory cytokines contribute to CFBD. However, epidemiological investigations and animal models also support a direct causal link between inactivation of skeletal cystic fibrosis transmembrane regulator (CFTR), the gene that when mutated causes CF, and CFBD. The objective of this study was to examine the direct actions of CFTR on bone. Expression analyses revealed that CFTR mRNA and protein were expressed in murine osteoblasts, but not in osteoclasts. Functional studies were then performed to investigate the direct actions of CFTR on osteoblasts using a CFTR knockout (*Cftr*−/−) mouse model. In the murine calvarial organ culture assay, *Cftr*−/− calvariae displayed significantly less bone formation and osteoblast numbers than calvariae harvested from wildtype (*Cftr*+/+) littermates. CFTR inactivation also reduced alkaline phosphatase expression in cultured murine calvarial osteoblasts. Although CFTR was not expressed in murine osteoclasts, significantly more osteoclasts formed in *Cftr*−/− compared to *Cftr*+/+ bone marrow cultures. Indirect regulation of osteoclastogenesis by the osteoblast through RANK/RANKL/OPG signaling was next examined. Although no difference in receptor activator of NF-κB ligand (*Rankl*) mRNA was detected, significantly less osteoprotegerin (*Opg*) was expressed in *Cftr*−/− compared to *Cftr*+/+ osteoblasts. Together, the *Rankl*:*Opg* ratio was significantly higher in *Cftr*−/− murine calvarial osteoblasts contributing to a higher osteoclastogenesis potential. The combined findings of reduced osteoblast differentiation and lower *Opg* expression suggested a possible defect in canonical Wnt signaling. In fact, Wnt3a and PTH-stimulated canonical Wnt signaling was defective in *Cftr*−/− murine calvarial osteoblasts. These results support that genetic inactivation of CFTR in osteoblasts contributes to low bone mass and that targeting osteoblasts may represent an effective strategy to treat CFBD.

## Introduction

Advances in cystic fibrosis (CF) care in the past 20 years have dramatically extended survival of CF patients, but age related complications such as low bone mass have emerged. The association of low bone mineral density (BMD) with CF was first reported in 1979 [1], [2]. In a large study of adult CF patients, 57% had osteoporosis that translated to a 100-fold greater risk of vertebral compression fracture [3]. Fractures not only cause pain and disability, but vertebral fractures and kyphosis further impair breathing biomechanics and often disqualify a patient from lung transplantation [4]. CF-related bone disease (CFBD) is the result of decreased osteoblast-dependent bone formation and increased osteoclastic bone resorption. This pattern of uncoupled bone turnover has been reported in both animal and clinical studies [5]–[9].

CFBD has been attributed to vitamin D deficiency, poor nutrition, hypogonadism, increase in inflammatory cytokines and glucocorticoid therapy. Other risk factors include male gender, advanced lung disease, malnutrition, and low fat-free body mass [10]. The F508del-CFTR (cystic fibrosis transmembrane conductance regulator) is the most frequent mutation that results in non-delivery of the protein to the cell membrane and confers an essentially null CFTR protein. This mutation compared to less severe CFTR mutations was also independently associated with reduced BMD in adult CF patients [10]. Whether these risk factors contribute indirectly to bone loss or CFTR mutations directly impact bone, has been a topic of controversy [11].

The skeletal phenotype of CF mouse models suggests that CFTR indeed has direct actions on bone. An advantage of CF mouse models in studying bone is the lack of CF-related complications found in human CF patients that influence BMD. CFTR knockout mice do not develop significant lung disease or a secondary increase in circulating inflammatory cytokines common in CF patients [9], [12], [13]. Proposed mechanisms for the lack of profound lung disease in CF mouse models include alternative chloride channels present in the mouse airway that compensate for CFTR absence and differences in bacterial airway colonization [14], [15]. Exocrine pancreatic insufficiency also does not occur in CF mouse models. Similarly, other non-CFTR chloride channels present in pancreatic ducts and acini may prevent insufficiency of the mouse exocrine pancreas and subsequent malnutrition, vitamin D deficiency and secondary hyperparathyroidism [14]. Although intestinal obstruction occurs in both humans and mice with defective CFTR, this alone would not contribute directly to CFBD. Despite the absence of these confounding factors, both CFTR knockout and F508del-CFTR mouse models recapitulate the human CFBD phenotype of reduced trabecular bone volume and cortical thickness, and impaired bone formation and enhanced bone resorption [8], [9], [12], [16]. Similarly, “gut corrected” CFTR knockout mice also demonstrate a similar bone phenotype further suggesting that CF bone disease is not solely due to intestinal obstruction [17].

We examined CFTR expression and determined the direct biological consequences of CFTR inactivation in murine bone using the knockout mouse model. CFTR was expressed in the osteoblast and inactivation resulted in defective differentiation, delayed new bone formation, and impaired osteoblast canonical Wnt signaling. Although CFTR was not expressed in osteoclasts, enhanced osteoclastogenesis is likely a direct effect of reduced osteoblast osteoprotegerin expression. This is the first report demonstrating direct *in vitro* effects of CFTR on osteoblasts. Defining the mechanisms of bone loss and determining optimal treatments are critical because CF patients are now living longer and the prevalence of fractures is expected to increase.

## Materials and Methods

### Animal use

Animal experimentation was approved by the University of Alabama at Birmingham Institutional Animal Care and Use Committee. Study mice with a global CFTR inactivation have been described previously [18], [19]. The colony was maintained by breeding *Cftr*+/− to *Cftr*+/+ C57BL/6 mice. *Cftr*−/− mice were generated by crossing *Cftr*+/− to *Cftr*+/− mice.

### Murine calvarial organ and primary osteoblast cultures

Calvariae were harvested from *Cftr*−/− and *Cftr*+/+ pups or from pups derived from WT timed-pregnant ICR Swiss female mice as previously described [20], [21]. Briefly, four-day-old pups were euthanized by CO_2_ inhalation. Calvariae were excised, cut along the sagittal suture, and each half placed on a stainless steel grid in a 12-well tissue culture plate containing 1 ml BGJ media (Life Technologies), 0.1% bovine serum albumin, 100 I.U./ml penicillin and 100 µg/ml streptomycin. After 14 days in culture, calvariae were fixed in 10% buffered formalin for 24 hours and decalcified with 10% EDTA for 48 hours. Tissue samples were then processed, paraffin-embedded, sectioned and stained with H&E/Orange-G. New bone area and osteoblast number were analyzed in quadruplicate samples using the BioQuant Image Analysis Software (BIOQUANT Image Analysis Cooperation, Nashville, TN). Histomorphometric analysis of calvariae was performed in quadruplicate using a 20X objective lens. New bone area and osteoblast number was determined from a calvarial microscopic field that measured 0.57 mm in length.

For primary osteoblast cultures, calvariae were washed in PBS and then placed in PBS (2 ml per mouse litter) containing 0.1% collagenase (Wako Pure Chemical Industries) and 0.2% dispase (Roche Applied Science). Calvariae were agitated at 37°C for seven minutes to release cells. The first extraction was discarded and the process was repeated three times. Osteoblasts isolated in the last three extractions were combined [22]. Cells were plated at a density of 10^6^ cells/ml in αMEM, 10% fetal calf serum, 100 I.U./ml penicillin, and 100 µg/ml streptomycin.

For osteoblast differentiation assays, primary osteoblast cultures were prepared from *Cftr*−/− and *Cftr*+/+ calvariae. Seven days after confluence, cells were washed, fixed in formalin for 30 minutes, washed again and stained for alkaline phosphatase using SigmaFast BCIP/NTB reagent (Sigma-Aldrich).

Proliferation of *Cftr*−/− and *Cftr*+/+ calvarial osteoblasts was assessed by bromodeoxyuridine (BrdU) staining. Briefly, 0.5×10^4^ *Cftr*−/− and *Cftr*+/+ calvarial osteoblasts were plated into each well of 96-well plate in quadruplicate. Three days after plating, BrdU was added according the manufacturers directions (Roche) and proliferation was determined by colorimetric assessment. Apoptosis was determined using a FITC-annexin V cell membrane detection kit (BD Pharmingen) with FACS analysis.

### Murine bone marrow osteoclast cultures

Bone marrow was collected from femurs and tibias of 8–9 week old *Cftr*−/− and *Cftr+/+* mice. Bone marrow cells were counted using a Coulter Z2 Particle Count and Size Analyzer (Beckman Coulter, Inc), excluding cells less than 7.5 µm in diameter. Cells were plated in quadruplicate at a density of 2×10^6^ cells/0.5 ml in each well of a 24-well plate. Bone marrow isolates were cultured in α-MEM containing L-glutamine, 100 IU/ml penicillin, 100 µg/ml streptomycin, 10% fetal bovine serum and 1×10^−8^ M 1,25(OH)_2_D_3_. Osteoclast cultures were stained on day 8 with a tartrate-resistant acid phosphatase (TRAP) kit (Sigma) and osteoclasts, TRAP-staining cells containing ≥3 nuclei, were counted via visual inspection by light microscopy.

### Immunohistochemistry

Calvariae were harvested from 4 day-old mouse litters containing *Cftr*−/− and *Cftr*+/+ pups. After confirmation of genotype, *Cftr*−/− and *Cftr*+/+ calvariae were fixed in 10% formalin and decalcified. Calvariae were processed, paraffin embedded and sectioned. Sections were blocked with 10 mg/ml BSA in PBS and incubated overnight at 4°C with the anti-CFTR 3G11 rat monoclonal antibody (Cystic Fibrosis Folding Consortium) at a 1∶500 dilution. After washing with PBS, sections were incubated with an anti-rat biotin-conjugated secondary antibody for 30 minutes. Vectastain ABC Reagent (Vector Laboratories) was used for detection. Sections were counterstained with hematoxylin.

### Immunofluorescence

Three-week-old *Cftr*−/− and *Cftr*+/+ bone marrow from tibiae, femora and humeri was flushed with αMEM, centrifuged, and plated at a density of 6×10^5^ cells/cm^2^ on collagen-coated cover slips in αMEM, 10% fetal calf serum, 100 I.U./ml penicillin and 100 µg/ml streptomycin. On day two, 10 ng/ml M-CSF (R&D Systems) and 30 ng/ml RANKL (R&D Systems) were added. On day 8, cells were fixed in 4% paraformaldehyde for 20 min at RT, washed with PBS and permeabilized with 0.1% Triton X-100 for 15 min at RT. After blocking with 10 mg/ml BSA (Sigma-Aldrich) in PBS, cells were incubated overnight at 4°C with the anti-CFTR 3G11 rat monoclonal (1∶500 dilution, Cystic Fibrosis Folding Consortium), and/or 3G11 and anti-NHERF (1∶500, Affinity Bioreagents) followed by incubation for 1 hour with Alexa 555 anti-rat IgG (1∶500, Life Technologies), and/or Alexa 488α-mouse IgG (1∶500, Life Technologies), and 4′,6-diamidino-2-phenylindole (DAPI) (300 nM, Life Technologies). Images were obtained using a Zeiss LSM 710 Laser confocal microscope system.

### Real-time PCR

Primary osteoblast cultures were washed in ice-cold PBS and RNA was extracted using the RNeasy Kit (Qiagen). A DNase digestion step was included during RNA purification. Real-time PCR (RT-PCR) was performed using the iQ SYBR Green Supermix and MyIQ Single-Color Real-Time PCR Detection System (BioRad). Changes in mRNA concentration were determined using the 2^−[Δ][Δ]Ct^ method and *Rpl32* as the internal control. Primer sequences were: *Cftr* F: ctcaggctccagcaatcttc, R: gcaccaaatcagcactagca; *Rpl32* F: cagggtgcggagaaggttcaaggg, R: cttagaggacacgttgtgagcaat; *Rankl* F: tgtactttcgagcgcagatg, R: cccacaatgtgttgcagttc; *Opg* F: gttcctgcacagcttcacaa, and R: aaacagcccagtgaccattc. Rankl and Opg expression was assessed after 24 hours of human parathyroid hormone (PTH) (1–34) 10 nM (American Peptide) treatment.

### Signaling assays

Cyclic AMP (cAMP) immunoassay (R&D Systems) was performed according to manufacturer's directions after treatment with human PTH (1–34) 10 nM for 10 minutes. Canonical Wnt activity was determined by transfecting calvarial osteoblasts with either the TOP-Flash or FOP-Flash Wnt luciferase reporter vectors (EMD Millipore) after 48 hours of treatment with human PTH (1–34) 10 nM and mouse Wnt3a 10 ng/ml (R&D Systems). Luciferase assays were performed using the Dual-Luciferase Reporter Assay (Promega, Madison, WI) and a BioTek Synergy 2 microplate reader (BioTek, Winooski, VT).

### Statistical analyses

Statistical analyses were performed using Prism 5.0 software. Comparisons of two groups were performed using an unpaired, two-tailed t test. Comparisons of three of more groups were performed using one-way ANOVA with Tukey post-test analysis. Significant differences are indicated (* = p<0.05; ** = p<0.01; *** = p<0.001).

## Results

### CFTR expression in bone

**Figure 1:**
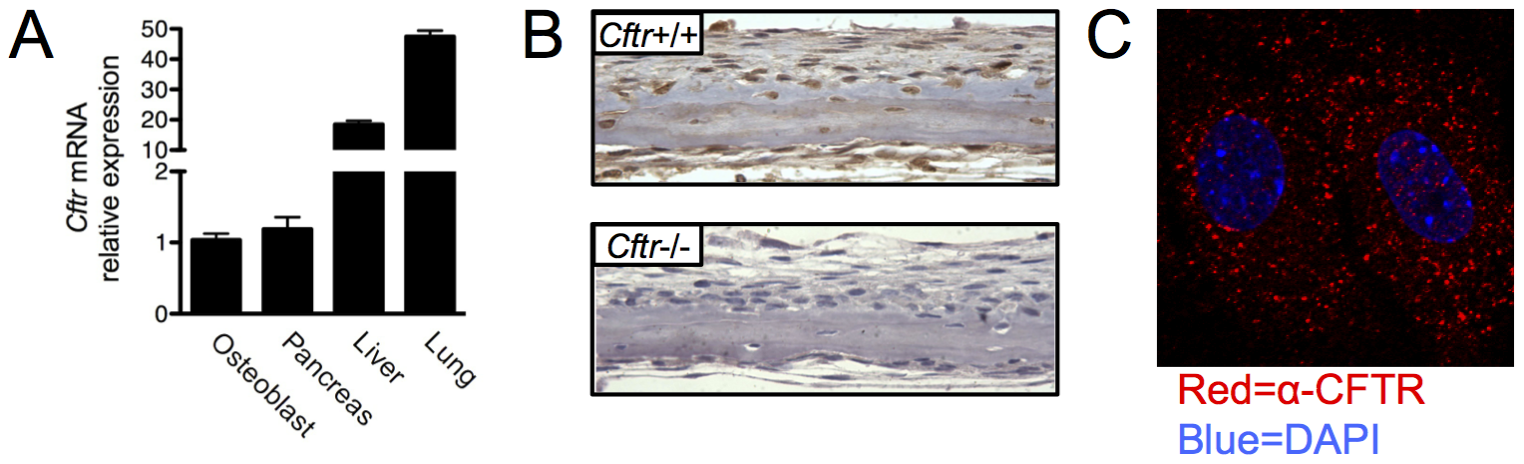
CFTR expression in osteoblasts. (A) Total RNA was harvested from mouse tissues. *Cftr* mRNA expression in murine primary osteoblast cultures, pancreas, liver and lung was assessed by real-time RT PCR. (B) Calvariae were harvested from *Cftr*+/+ and *Cftr*−/− 4-day-old pups, fixed, sectioned immunostained for CFTR using the 3G11 rat anti-CFTR monoclonal antibody and counterstained with hematoxylin. (C) Osteoblasts were prepared by digestion from murine calvariae and cultured on collagen-coated glass slides. CFTR (red) was identified by immunofluorescence using the 3G11 antibody. Nuclei (blue) were identified by DAPI staining. Two adjacent osteoblasts are shown.

Initial studies were directed at determining CFTR expression in mouse bone cells. *Cftr* mRNA was detected in calvarial osteoblasts as well as murine liver, lung and pancreas (Figure 1A). Comparable pancreas and osteoblast mRNA concentration suggested that the degree of *Cftr* expression in the osteoblast was physiologically relevant since *Cftr* inactivation also results in pancreatic disease. *In vivo* protein expression was next examined utilizing neonatal murine calvarial organ cultures, a rich source of osteoblasts. CFTR protein was detected in wild type (*Cftr*+/+) but not in CFTR knockout (*Cftr*−/−) calvariae by immunostaining using the 3G11 rat monoclonal anti-CFTR antibody (Figure 1B). Osteoblasts and osteocytes embedded within bone displayed similar expression. In a recent report, the 3G11 antibody displayed excellent CFTR detection specificity compared to other antibodies that reportedly detect mouse CFTR in a panel of Cftr+/+ and Cftr−/− skeletal tissues [23]. Immunofluorescence of cultured murine calvarial osteoblasts revealed abundant punctate intracellular staining (Figure 1C).

**Figure 2:**
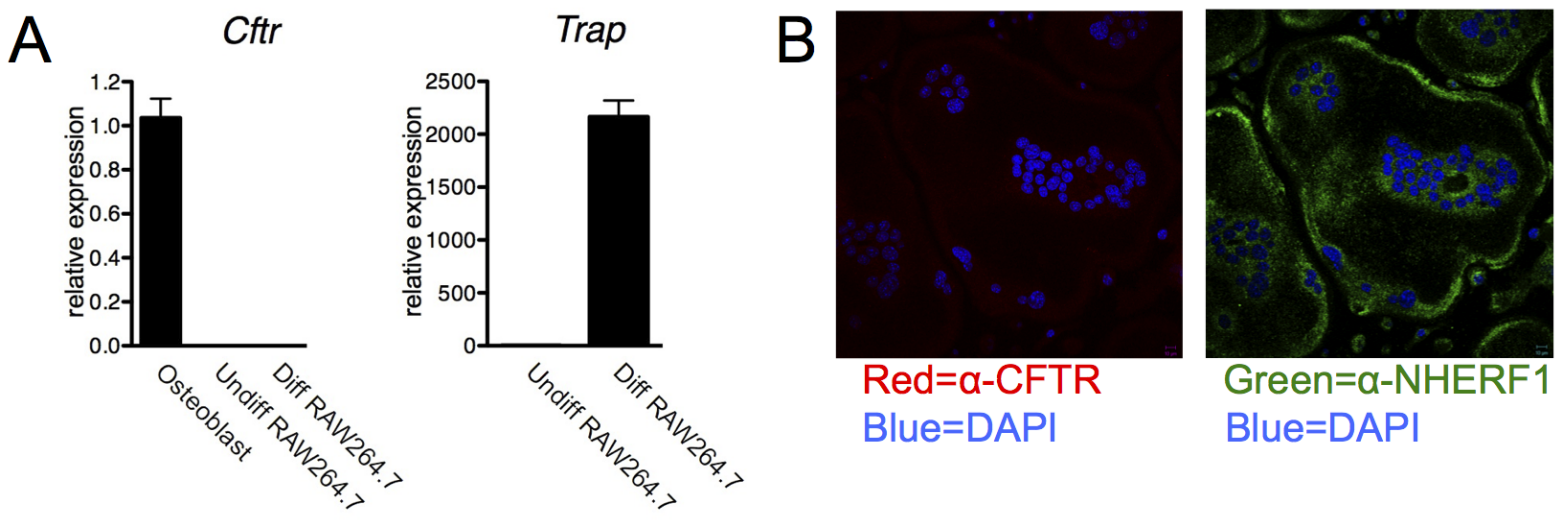
Lack of CFTR expression in osteoclasts. (A) *Cftr* mRNA expression analysis of calvarial osteoblasts, undifferentiated and osteoclast-differentiated (M-CSF/RANKL-treated) RAW264.7 murine monocyte cells. *Trap* mRNA expression served as a control for osteoclast formation. (B) Immunofluorescence did not reveal CFTR (red) expression in murine bone marrow-derived multinucleated osteoclasts. Membrane-localized protein Na^+^/H^+^ exchange regulatory cofactor-1 (NHERF1) (green) served as a positive control for membrane staining. Nuclei (blue) was identified by DAPI staining.

Osteoclast *Cftr* expression was next examined. *Cftr* mRNA was not detected in the mouse macrophage cell line RAW 264.7 (Figure 2A). These osteoclast precursor cells were then treated with macrophage colony-stimulating factor (M-CSF) and receptor activator of NF-κB ligand (RANKL) to induce osteoclastogenesis. Message for TRAP, a specific osteoclast marker, was detected in treated, but not untreated, RAW 264.7 cells indicating successful osteoclast differentiation. Despite differentiation with M-CSF and RANKL, *Cftr* mRNA remained undetectable after 35 PCR cycles (Figure 2A). To confirm the absence of expression, CFTR protein was not detected in osteoclasts derived from mouse bone marrow by immunofluorescence staining (Figure 2B). An antibody to the membrane protein Na^+^/H^+^ exchange regulatory cofactor-1 (NHERF) served as a control for osteoclast membrane staining.

### CFTR osteoblast actions

**Figure 3:**
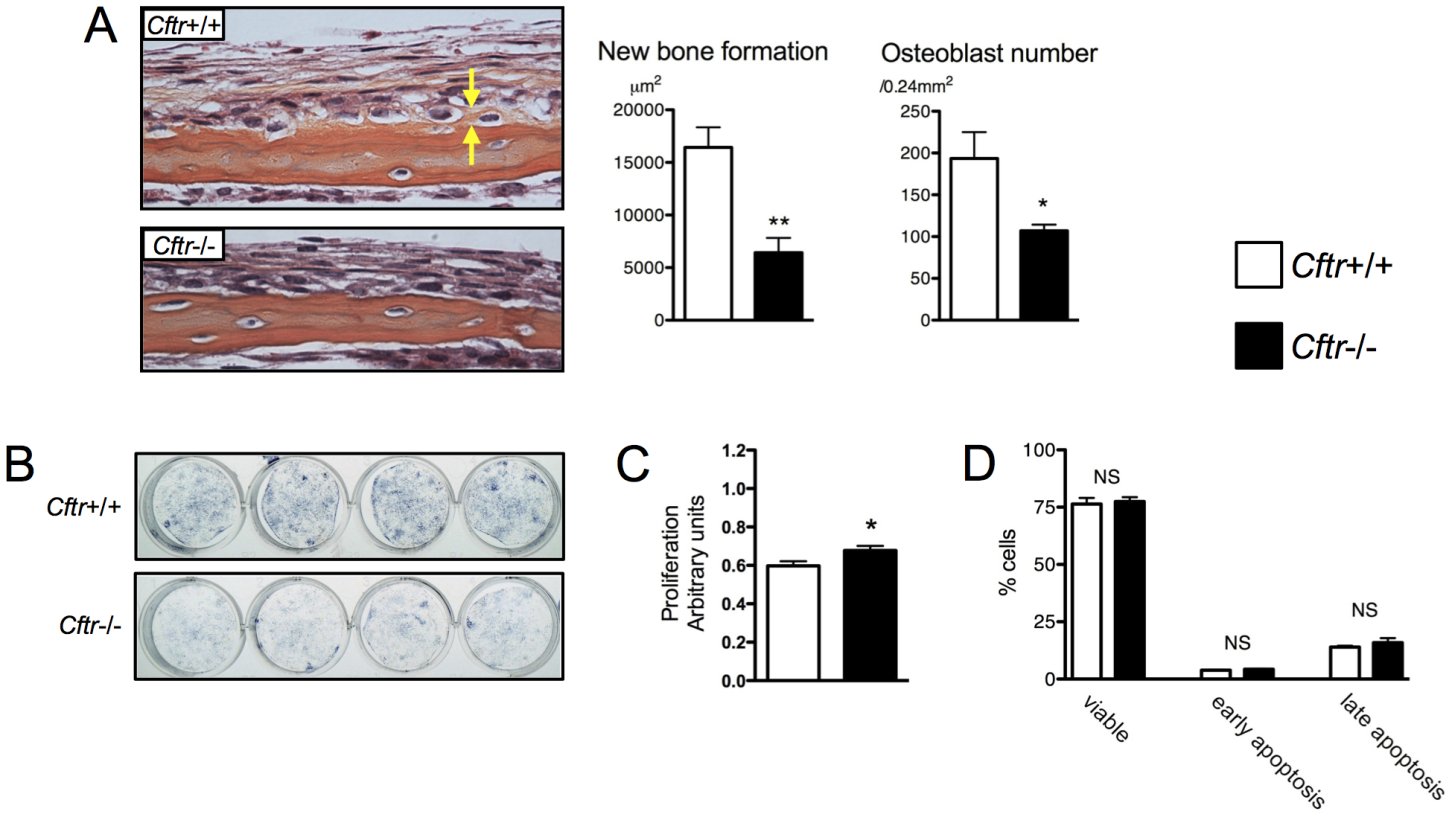
CFTR inactivation reduced osteoblast differentiation and bone formation. (A) The murine calvarial organ culture assay was performed on *Cftr*+/+ and *Cftr*−/− mouse pups. After two weeks in culture, new bone formation and osteoblast number was significantly less with *Cftr* inactivation. Yellow arrows indicate the area of actively mineralizing bone indicated by orange G staining that is distinct from the red staining of eosin. (B) Murine calvarial osteoblasts harvested from *Cftr*+/+ and *Cftr*−/− calvariae were grown in culture. Seven days after reaching confluence, osteoblast differentiation was assessed by alkaline phosphatase staining. (C) Proliferation, as assessed by BrdU staining, was modestly increased in *Cftr*+/+ compared to *Cftr*−/− calvarial osteoblasts. (D) The proportion of calvarial osteoblasts that were viable, undergoing early apoptosis or late apoptosis was unchanged with CFTR inactivation. (* = p<0.05; ** = p<0.01)

The consequence of restricted osteoblast CFTR expression in bone was investigated. The murine calvarial organ culture assay is a reliable method to measure osteoblast activity and bone formation [20], [21]. Calvariae were harvested from *Cftr*−/− and *Cftr*+/+ 4 day-old pups and the calvarial organ culture bone formation assay was performed. After 14 days in culture, *Cftr*−/− calvariae displayed significantly less new bone (6430 vs. 16440 µm^2^, p = 0.0023) and fewer osteoblasts (109 vs. 194 Obl/0.24 mm^2^, p = 0.04) than *Cftr*+/+ calvariae (Figure 3A). The *in vitro* effects of osteoblast CFTR inactivation were also examined. Calvarial osteoblasts harvested from *Cftr*+/+ and *Cftr*−/− littermates were plated at the same density and grown in cell culture for seven days after achieving confluence. Osteoblast cultures were stained for alkaline phosphatase, a marker of differentiation. Significantly less staining was observed in *Cftr*−/− osteoblasts indicating a defect in differentiation (Figure 3B). An expected consequence of delayed differentiation was a slight increase in the proliferative rate (Figure 3C). Osteoblast apoptosis was unaffected by CFTR inactivation (Figure 3D).

### Indirect effects of CFTR on osteoclastogenesis

**Figure 4:**
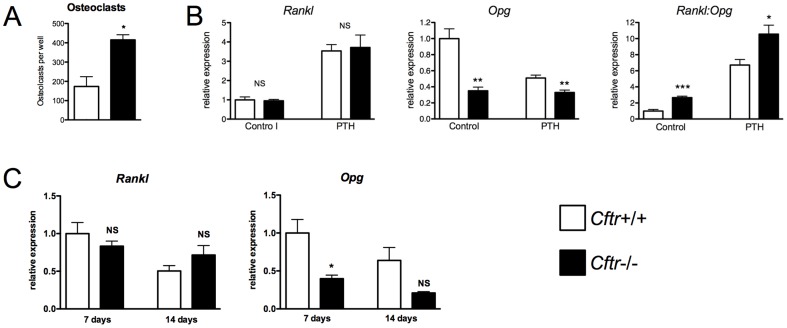
CFTR inactivation increased osteoclastogenesis and *Rankl*:*Opg* osteoblast expression. (A) Bone marrow was flushed from *Cftr*+/+ and *Cftr*−/− mice and osteoclast formation assays performed. Osteoclasts were identified by TRAP staining as cells containing ≥3 or more nuclei. More osteoclasts were found in *Cftr*−/− bone marrow. (B) *Rankl* and *Opg* mRNA expression was determined by real-time RT PCR in *Cftr*+/+ and *Cftr*−/− calvarial osteoblasts with and without PTH (1–34) 10 nM treatment for 24 hours. Overall, CFTR inactivation increased the *Rankl*:*Opg* ratio in both PTH (1–34)-treated and untreated osteoblasts. (C) In long-term osteoblast cultures, decreased *Opg* expression persisted post-confluence despite no significant difference in *Rankl*. *Opg* expression remained significantly lower at 7 days in *Cftr*−/− osteoblasts, but did not reach significance at 14 days post-confluence. (* = p<0.05; ** = p<0.01; *** = p<0.001; NS  =  not significant)

Despite the lack of CFTR osteoclast expression, CF patients and CF animal models demonstrate enhanced osteoclastic bone resorption. An *in vitro* osteoclastogenesis assay was performed on bone marrow harvested from *Cftr*+/+ and *Cftr*−/− mice. This osteoclast formation assay relies on the presence of bone marrow-derived osteoblasts and stromal cells to provide M-CSF and RANKL and to drive osteoclastogenesis. Significantly more TRAP-positive osteoclasts were found in the *Cftr*−/− bone marrow cultures compared to cultures harvested from *Cftr*+/+ mice (Figure 4A).

Critical signaling molecules that regulate osteoclastogenesis are RANKL and Osteoprotegerin (OPG). OPG, a member of the tumor necrosis factor (TNF) receptor superfamily, is a secreted RANKL decoy receptor of osteoblastic-lineage cells [24], [25]. OPG achieves its effect on osteoclasts indirectly by binding to, and blocking the effect of RANKL [26]. Therefore, the ratio of RANKL to OPG determines osteoclast formation potential.

The effects of CFTR inactivation on osteoblast *Rankl* and *Opg* expression were examined to uncover mechanisms of enhanced bone resorption. Subconfluent *Cftr*+/+ and *Cftr*−/− osteoblasts were treated with and without PTH (1–34) for 24 hours, and *Rankl* and *Opg* mRNA was measured (Figure 4B). PTH was used as a control to increase *Rankl* and decrease *Opg* osteoblast expression [24], [27]–[29]. An equivalent amount of *Rankl* mRNA was detected in *Cftr*+/+ and *Cftr*−/− osteoblasts. PTH (1–34) expectedly increased *Rankl* mRNA to a similar degree in both groups. However, CFTR inactivation led to significantly less *Opg* expression. PTH (1–34) did not alter *Opg* mRNA in *Cftr*−/− osteoblasts but expectedly reduced mRNA concentration in *Cftr*+/+ osteoblasts. Overall, the *Rankl*:*Opg* ratio was higher in *Cftr*−/− compared to *Cftr*+/+ osteoblasts with or without PTH (Figure 4B). These data suggested that increased osteoclastic bone resorption with CFTR inactivation in humans and mice may be due to enhanced osteoblast-mediated osteoclastogenesis. A follow-up experiment examined the durability of these data in osteoblasts cultured 7 and 14 days after reaching confluence. *Opg* expression remained significantly lower at 7 days in *Cftr*−/− osteoblasts, but did not reach significance at 14 days post-confluence (Figure 4C). No significant difference in *Rankl* between the *Cftr*+/+ and the *Cftr*−/− osteoblasts cultured for 7 or 14 days post-confluence was detected.

**Figure 5:**
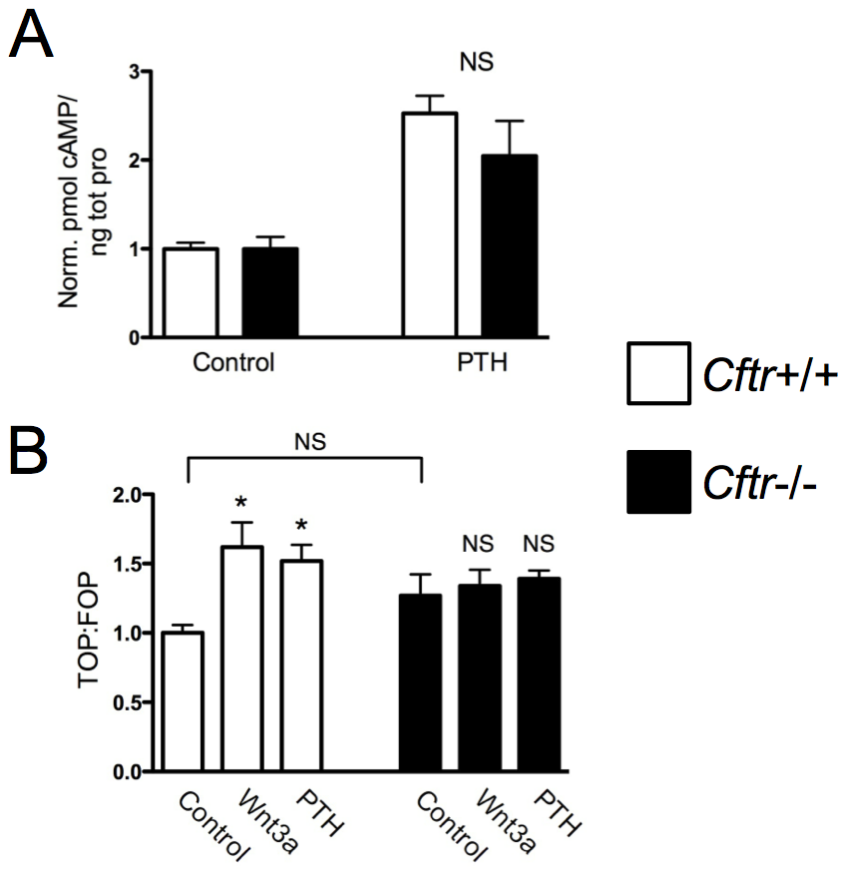
CFTR inactivation blocked Wnt3a and PTH-activated osteoblast canonical Wnt signaling. (A) Calvarial osteoblasts harvested from *Cftr*+/+ and *Cftr*−/− mouse pups were cultured. Cyclic AMP accumulation, a marker of PTH receptor activation, was not significantly different between *Cftr*+/+ and *Cftr*−/− osteoblasts after 10 minutes of PTH (1–34) 10 nM treatment. (B) Activation of canonical Wnt signaling was assessed utilizing the TOP-Flash and FOP-Flash Wnt luciferase reporter vectors. Canonical Wnt activity increased after 48 hours of PTH (1–34) 10 nM and Wnt3a 10 ng/ml treatment in *Cftr*+/+ but not in *Cftr*−/− calvarial osteoblasts. (* = p<0.05; NS  =  not significant)

Cyclic AMP (cAMP) mediates PTH-stimulated reduction in *Opg* and increase in *Rankl* expression via coupling of the G-protein coupled receptor PTH1R to G-protein Gα_s_ [28]. Differences in cAMP cellular accumulation with PTH in *Cftr*+/+ and *Cftr*−/− osteoblasts were measured. A brief 10 minute PTH (1–34) treatment increased cAMP approximately two-fold in both *Cftr*+/+ and *Cftr*−/− osteoblasts suggesting that the absence of osteoblast CFTR does not alter PTH-induced cAMP accumulation (Figure 5A).

Another critical signaling pathway that regulates both osteoblast differentiation and *Opg* expression is canonical Wnt signaling [30], [31]. Forty-eight hours of Wnt3a and PTH treatment expectedly increased canonical Wnt activity in *Cftr*+/+ osteoblasts but not in *Cftr*−/− osteoblasts (Figure 5B). These data suggest that dysfunctional canonical Wnt signaling contributes to the uncoupled bone phenotype characteristic of CF.

## Discussion

The average life expectancy of CF patients 60 years ago was less than 5 years, but treatment advances have extended the average life expectancy now to 36.8 years [32]. It is expected that continued progress in this area will result in even greater longevity. Because CF patients are now older, other age-related complications have emerged such as clinically significant fractures of the spine, hip and forearm [3]. Malnutrition, glucocorticoids, chronic inflammation and hypogonadism individually contribute to reduced bone accrual and low BMD in CF patients. Clinical observations and animal models of CF bone disease hint that CFTR mutation in bone also impacts bone remodeling [5]–[9]. The results reported here support a CF disease model in which CFTR expression in bone directs reduced osteoblast differentiation and enhanced osteoclastic bone resorption.

The most recognizable CF complications are the result of CFTR dysfunction in endoderm-derived secretory epithelial cells. This report and other recent studies demonstrate that CFTR is expressed in mesenchyme-derived osteoblasts, odontoblasts, chondrocytes and myocytes and that mutations directly perturb cellular activities of these cell types [23], [33]–[35]. Mechanisms of CFTR action in mesenchymal cells likely differ from the well-recognized actions in epithelial cells. In respiratory epithelium, CFTR is located almost exclusively within the apical surface and cooperates with other chloride channels located on the basolateral surface and intercellular tight junctions to move chloride ions towards the apical cell surface. Osteoblasts and other mesenchymal-derived cells are not arranged in a strictly apical-basolateral orientation, and do not form conductance barriers when grown in monolayers. The scarcity of CFTR within the osteoblast cell membrane reported here, as contrasted by the prominent cell membrane location of epithelial cells, supports this conclusion. The prominent intracellular location of CFTR has also been reported in human osteoblasts and dentin-forming odontoblasts [23]. It is unclear as to the intracellular function of CFTR but some have proposed involvement in phagosome acidification and in sarcoplasmic reticulum calcium homeostasis of skeletal muscle [23], [35], [36]. CFTR is a large membrane protein with multiple binding domains that associate with the Na^+^/H^+^ exchanger regulatory factors-1 and -2 (NHERFs) and other binding partners [37]–[39]. Other investigators have also speculated that CFTR may also act as a scaffold protein to anchor other membrane proteins and assist in proper cell signaling.

CFTR was expressed at both the message and protein level in osteoblasts but absent in osteoclasts. A study examining CFTR expression in human bone detected CFTR in both osteoblasts and osteoclasts by immunostaining [40]. However, the antibody used in that study was recently reported to have poor antigen specificity [23]. A survey of commonly used CFTR antibodies reported that the 3G11 antibody used in this study was specific for the CFTR protein as evidenced by the lack of staining in tissues derived from CFTR knockout mice [23]. Of course, differences in CFTR expression between humans and mice could explain this discrepancy in osteoclast CFTR expression. Because of the mouse expression data reported here, studies to uncover CFTR function in bone were directed towards the osteoblast.

Osteoblast CFTR inactivation reduced *Opg* but not *Rankl* message. This result is consistent with other reports of reduced OPG in CF bone disease [41], [42]. The unique pattern of delayed osteoblast differentiation and downregulated *Opg* expression is similar to reports of mice with aberrant osteoblast canonical Wnt signaling. Mice with genetic inactivation of the Wnt target β-catenin had reduced trabecular and cortical bone volume due to delayed osteoblast differentiation [31], [43]. Osteoclast number in bone was also increased because of reduced OPG [31], [43]. In *Cftr*−/−osteoblasts, the lack of Wnt signaling activation with either PTH or Wnt3a supports dysregulated canonical Wnt signaling. However, canonical Wnt signaling also downregulates RANKL expression [43]–[45]. In the absence of active canonical Wnt signaling, PTH may therefore have been expected to increase RANKL expression even more with CFTR inactivation due to unopposed PTH/cAMP/PKA signaling. Since this was not observed, CFTR inactivation may have additional effects on pathways outside of canonical Wnt signaling. The *in vitro* evidence of delayed osteoblast new bone formation and reduced OPG expression potentially directing enhanced osteoclastic bone resorption parallels the uncoupled bone turnover that is characteristic of CFBD in humans and animal models of CF bone disease.

These data have important implications for optimizing CF bone disease treatment. In addition to vitamin D, calcium supplements, and nutritional support, adult CF patients with T- or Z-scores on DXA scans equal to or less than −2.0 should be offered bisphosphonate therapy [11]. Teriparatide (PTH 1–34) is the only drug currently available that stimulates osteoblast bone formation and is superior to bisphosphonates in increasing BMD and reducing skeletal fractures in postmenopausal women [46]. Our data suggest that PTH does not activate canonical Wnt signaling and therefore teriparatide my have blunted bone anabolic effects in CF patients. Denosumab, a recently available antiresorptive medication that targets RANKL, may also be particular effective in CF patients to counteract an increased osteoblast RANKL:OPG ratio. Future trials are therefore needed to address optimal treatment regimens for treating CF bone disease. A detailed understanding of the pathogenesis of CF bone disease and the role of CFTR in bone cell function is clearly important to reduce skeletal fractures.
